# Time-related performance characteristics of high-sensitivity troponin I assay using manufacturer’s controls and reagents

**DOI:** 10.1016/j.plabm.2021.e00217

**Published:** 2021-03-26

**Authors:** Rosanna Inzitari, Sebastian Vencken, David Daghfal, Karl McAuley, Anthony McDermott, Marie Galligan, Peter Doran

**Affiliations:** a Clinical Research Centre, UCD School of Medicine, University College Dublin, Dublin, Ireland; b Diagnostics Division, Abbott Laboratories, Abbott Park, IL, USA

**Keywords:** Troponin, Cardiovascular disease, Diagnostics, Assay, Immunoassay

## Abstract

**Background:**

Troponin is a widely used cardiac protein biomarker for acute coronary syndrome. Its increasing importance drives an increasing need to assess, in real-world conditions, the performance of the tests to measure it. We evaluated the performance characteristics of high-sensitivity troponin I assay reagents and ancillary agents on the Abbott ARCHITECT ci4100, ARCHITECT i2000SR and Alinity ci using historical quality control data spanning 5 years.

**Design and method:**

Retrospective diagnostic hs-TnI quality control data were collected between 2015 and 2019 from the Abbott ARCHITECT ci4100, ARCHITECT i2000SR and Alinity ci located in the University College Dublin Clinical Research Centre Core Lab facility. Descriptive statistics for bias and variability were generated. Linear regression models were used to calculate the mean hs-TnI concentrations over Abbott quality control or reagent lot age and over time from the last calibration of the analysers.

**Results:**

Measurement bias on all three systems ranged between -2.49% and 3.98%. The total CV was ≤8.80%, with a within-lot variability for the reagents and controls of ≤5.45% and ≤7.13%, respectively. The between-lot CVs for reagents and controls were ≤7.16% and 6.19%, respectively. The effect of control or reagent age did not greatly affect stability over time. Results were also stable over different times from the last calibration of the analysers.

**Conclusion:**

The results of this study show that the Abbott hs-TnI assay on the ARCHITECT i2000sr and ARCHITECT ci4000 systems is quite stable in a core laboratory environment. The Alinity ci system exhibits similar performance characteristics.

## Highlights

• Low variability and bias for a high-sensitivity troponin I assay on three Abbott analysers in a core laboratory setting.
• Performance of the high-sensitivity troponin I assay is stable accross the manufacturer’s specified shelf life and over time.
• The manufacturer’s specified calibration window is sufficient for stable high-sensitivity troponin I assay performance.

## 1 Introduction

Troponin is a cardiac protein biomarker that is commonly measured in the blood for the diagnosis and confirmation of acute coronary syndrome (ACS) [1]. The detection of this cardiac-specific biomarker has improved over time, allowing for higher sensitivity and specificity of analysis. The clinical usage has also evolved to current day use in early rule-out (e.g. studies evaluated cut-offs ranging from 2 to 5 ​ng/L with excellent NPV’s) [[2], [3], [4], [5], [6], [7]] and rule-in algorithms involving changes in troponin levels over 1–3 ​h periods of time and/or various low-level cut points such as the sex-specific 99^th^ percentiles of the populations (16–34 ​ng/L) [[8], [9], [10]]. Beyond these uses for patients with suspected ACS comes the more challenging use of this biomarker in risk stratification of asymptomatic patients and apparently healthy individuals [11]. Some studies have also indicated the usefulness of troponin measurements in monitoring patients post-non cardiac surgical procedures, or with heart failure [12,13]. Furthermore, troponin has also been identified as a useful biomarker for cancer chemotherapy-induced cardiac injury [14].

An increasing importance of troponin as a biomarker drives an increasing need for careful quality control assessments of the biomedical assays that measure it. Preferably, these quality control assessments should span over more extended periods of time and should cover the use of the assays in a biomedical laboratory setting. The lot-to-lot consistency of both assay reagents and the ancillary agents provided by the manufacturers, such as quality control materials and calibrators, is critical to ensuring stable results and timely reporting [15]. Performance consistency within the recommended shelf-life of these agents should also be assessed as small shelf-life-dependent changes may have implications to clinical risk levels and therefore drive treatment decisions.

Few studies have evaluated biological variability of troponin as a component in the overall total error of measurement equations [16,17]. Others have studied sigma metrics looking at bias and imprecision at specific decision thresholds [18]. Using historical assay data gathered from three clinical diagnostic systems for troponin I (TnI) measurements, the present study aims to retrospectively analyse the performance characteristics of high-sensitivity troponin I (hs-TnI) assay reagents and ancillary agents, including the effect of the age of these materials on these characteristics.

## 2 Methods and materials

### 2.1 Quality control workflow and retrospective data

Retrospective diagnostic hs-TnI quality control data were collected between 2015 and 2019 from the ARCHITECT ci4100, ARCHITECT i2000SR and Alinity ci (*Abbott Laboratories, IL, USA*) located and used in the Core Lab Facility based in the University College Dublin Clinical Research Centre, St. Vincent’s University Hospital, Dublin, Ireland. These systems are widely used as part of automated diagnostic workflows in biomedical laboratories worldwide. The Abbott ARCHITECT ci System is an integrated platform designed to perform automated chemistry tests, utilizing photometry and potentiometric technology and immunoassay tests, utilizing chemiluminescent microparticle immunoassay (CMIA) detection technology. The ARCHITECT i1000SR and i2000SR immunodiagnostic analysers deliver stat and routine results as needed with flexible random-access protocols. The more recent Alinity ci system is a more compact, functionally integrated system with higher capacity. It has features that allow for continuous access to reagents, samples, calibrators, controls and bulk solutions. The hsTnI assay is a two-step immunoassay using CMIA technology with flexible assay protocols, referred to as Chemiflex. It requires 210 ​μL of sample and has an 18 ​min processing time to first result.

The data for this study was gathered from quality control measurements that were part of the routine use of the system during clinical research studies. Manufacturer’s instructions were followed and implemented throughout all testing and quality control measurements were run daily on each of the three instruments. The hs-TnI assay reagents, henceforth referred to as ‘reagents’, used in this study were the STAT High Sensitive Troponin-I Reagent Kit *(Abbott Laboratories*) with the product code *3P25* for the ARCHITECT ci4100 and ARCHITECT i2000SR, and with the product code *08P13* for the Alinity ci.

The quality control materials, henceforth referred to as ‘controls’, used in this study were the STAT High Sensitive Troponin-I Controls (*Abbott Laboratories*) with the product code *3P25-11* for the ARCHITECT ci4100 and ARCHITECT i2000SR, and with the product code *08P13-10* for the Alinity ci. The controls exist at three different TnI concentrations (Low: 20 ​ng/L, Medium: 200 ​ng/L and High: 15,000 ​ng/L). The Low control is standardized to NIST per manufacturer’s package insert. The controls were assayed 4 times in duplicate on designated days at 2-h intervals to assess assay precision prior to study analysis, and one time in duplicate on every day the systems were used for diagnostic testing of TnI as a means of run validation. In total, 30 different hs-TnI reagent lots and 17 different control lots were used over the 5-year period. Lot numbers for the reagents and controls used in this study are reported in Table S1 (Supplementary File 1).

The calibrator materials used in this study were the STAT High Sensitive Troponin-I Calibrators (*Abbott Laboratories*) with the product code *3P25-02* for the ARCHITECT ci4100 and ARCHITECT i2000SR, and with the product code *08P13-01* for the Alinity ci.

The controls were assayed before the start of every study single study run between 2015 and 2019. In accordance with the CRC Core Lab SOP in adherence the package insert for the hsTnI the controls were run four times in duplicate on designated days at 2-h intervals to assess assay precision prior to study analysis, and one time in duplicate on every day the systems were used for samples testing of TnI as a means of run validation. Results were reviewed daily and range of validity was considered (2 standard deviations) before samples analysis. If measurements from controls deviated from the accepted range, a second run of all the control was performed. If the issue persisted, calibration was repeated before running the control samples. Each day, clinical measurements were performed conditional on the results from control samples.

### 2.2 Analysis

Bias in measurements was calculated as the mean percentage deviation from the manufacturer-specified control target concentrations (Bias_Target_) and from the factory release testing measurements (Bias_FRT_). Measurement variability of both hs-TnI reagents and control materials are reported as percent coefficients of variation (CV). For both reagents and controls, within-lot CVs (CV_intrareagent_ and CV_intracontrol_ respectively) and between-lot CVs (CV_interreagents_ and CV_intercontrol_ respectively) were calculated. Total CVs (CV_Total_) comprised all within- and between-lot reagent and control variability.

Linear regression models were used to calculate the mean hs-TnI concentrations over lot age and over the time from the last calibration. The equations for these models can be found in the Supplementary Methods (Supplementary File 1) as Eq (1) and Eq (2) respectively. Data analysis was performed using R 3.6.2 (R Core Team, 2019). The code and data used for this analysis can be found in Supplementary File 2 and Supplementary File 3, respectively.

## 3 Results

### 3.1 Bias and precision of control and reagent lots for hs-TnI

Descriptive statistics (sample size, mean, bias and CV) at the three TnI control concentration levels for the ARCHITECT ci4100, ARCHITECT i2000SR and Alinity ci are shown in Table 1. Although 13 reagent lots were run on the ARCHITECT ci4100, for two of these lots a field safety notice was issued for a product recall (see Table S1; Supplementary File 1). Hence, the data for these lots were not included in any of the results.

**Table 1:** Descriptive statistics for hs-TnI reagent lots and control lots on ARCHITECT ci4100, ARCHITECT i2000SR and Alinity ci.

ARCHITECT ci4100			
Statistic	Low (20 ​ng/L)	Medium (200 ​ng/L)	High (15,000 ​ng/L)
N_Reagent_	11	11	11
N_Control_	7	7	7
N_Total_	248	248	248
Mean *ng/L*	20.80	195.0	15,528
Bias_Target_*%*	3.98	−2.49	3.52
Bias_FRT_*%*	2.60	1.14	−0.08
CV_Intrareagents_*%*	5.25	3.84	4.35
CV_Interreagents_*%*	7.16	5.83	4.25
CV_Intracontrol_*%*	6.88	5.30	5.11
CV_Intercontrol_*%*	3.17	3.63	1.95
CV_Total_*%*	7.53	6.37	5.28
**ARCHITECT i2000SR**			
**Statistic**	**Low (20 ​ng/L)**	**Medium (200 ​ng/L)**	**High (15,000 ​ng/L)**
N_Reagent_	16	16	16
N_Control_	6	6	6
N_Total_	394	395	395
Mean *ng/L*	19.61	196.6	15,150
Bias_Target_*%*	−1.95	−1.72	1.00
Bias_FRT_*%*	0.37	−0.81	−2.03
CV_Intrareagents_*%*	5.45	4.09	3.09
CV_Interreagents_*%*	6.50	3.86	5.80
CV_Intracontrol_*%*	7.13	5.25	5.47
CV_Intercontrol_*%*	6.19	2.73	5.67
CV_Total_*%*	8.80	5.79	6.25
**Alinity ci**			
**Statistic**	**Low (20 ​ng/L)**	**Medium (200 ​ng/L)**	**High (15,000 ​ng/L)**
N_Reagent_	3	3	3
N_Control_	3	3	3
N_Total_	107	108	108
Mean *ng/L*	19.87	198.7	15,383
Bias_Target_*%*	−0.64	−0.67	2.55
Bias_FRT_*%*	−4.37	−0.94	2.37
CV_Intrareagents_*%*	5.44	4.59	4.02
CV_Interreagents_*%*	3.24	3.34	4.22
CV_Intracontrol_*%*	5.33	4.55	3.94
CV_Intercontrol_*%*	3.61	3.36	4.27
CV_Total_*%*	5.91	5.39	5.26

The number of reagent and control lots is expressed as N_Reagents_ and N_Control_ respectively. The total number of individual measurements performed is expressed as N_Total_.

The Bias_Target_ provides a measure of mean bias from the ideal target concentrations of the controls as stated in the header of Table 1. Bias_Target_ on all three systems ranged between -2.49% and 3.98%. Bias_FRT_ provides a measure of mean bias from the factory release testing of the controls by the manufacturer. Bias_FRT_ was between −4.37% and 2.60% on all three systems. The ARCHITECT i2000SR measurements showed the smallest (closest to 0%) overall Bias_Target_ and Bias_FRT_.

Total variability between runs is expressed as CV_Total_. On all the systems, the CV_Total_ was 8.80%, with the Alinity ci producing the lowest overall CV_Total_.

Total variability can be broken down in within-lot and between-lot variability. Within-lot variability for hs-TnI reagent lots was expressed as a pooled CV, weighted by the number of intra-lot replicates (CV_Intrareagents_). The CV_Intrareagents_ was 5.45% for all three systems, with the lowest reported by the ARCHITECT i2000SR. The variability within the hs-TnI control lots was similarly calculated (CV_Intracontrol_) and was ≤7.13% for all three systems, with the lowest reported by the Alinity ci.

The between-lot CVs for reagents (CV_Interreagents_) and controls (CV_Intercontrol_) were ≤7.16% and ≤6.19% respectively for all systems. The Alinity ci showed the lowest overall CV_Interreagents_ and the ARCHITECT ci4100 showed the lowest overall CV_Interreagents_.

The ARCHITECT ci4100 CV_Interreagents_ was higher than the CV_Intrareagents_ at all three TnI concentration levels. Similarly, this also occurred for the ARCHITECT i2000SR at Low and High TnI concentrations and for the Alinity ci High TnI concentration. Furthermore, CV_intracontrol_ was higher than CV_intercontrol_ for several TnI concentration levels on some systems. Typically for biomarker assays, intra-lot variability tends to be lower than inter-lot variability. However, in this study the higher intra-lot variability may possibly be explained by a variability component introduced from the use of the same reagent lot at multiple time points, while mean measurement of each lot, which contributes to inter-lot variability, is more stable.

Table 1 summarises all aggregate results from the three systems irrespective of the age of the hs-TnI reagents or controls. To control for reagents and control age, a second descriptive statistics table (Table S2; Supplementary File 1) was generated that included only the values from reagents and controls with the same maximum age for all three systems.

**Fig. 1:**
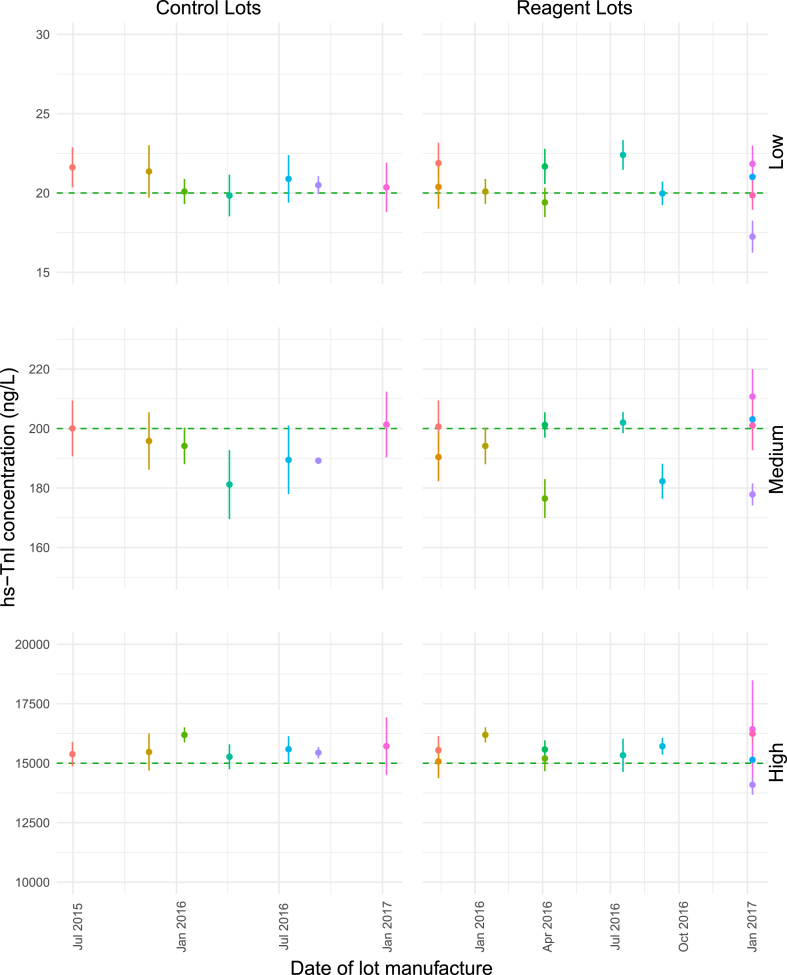
**Lot-specific mean hs-TnI concentrations over lot manufacturing date on the ARCHITECT ci4100**. Points indicate lot-specific means Each point is a unique lot. Error bars represent lot-specific standard deviations. Horizontal dashed line represents the ideal target hs-TnI concentration.

**Fig. 2:**
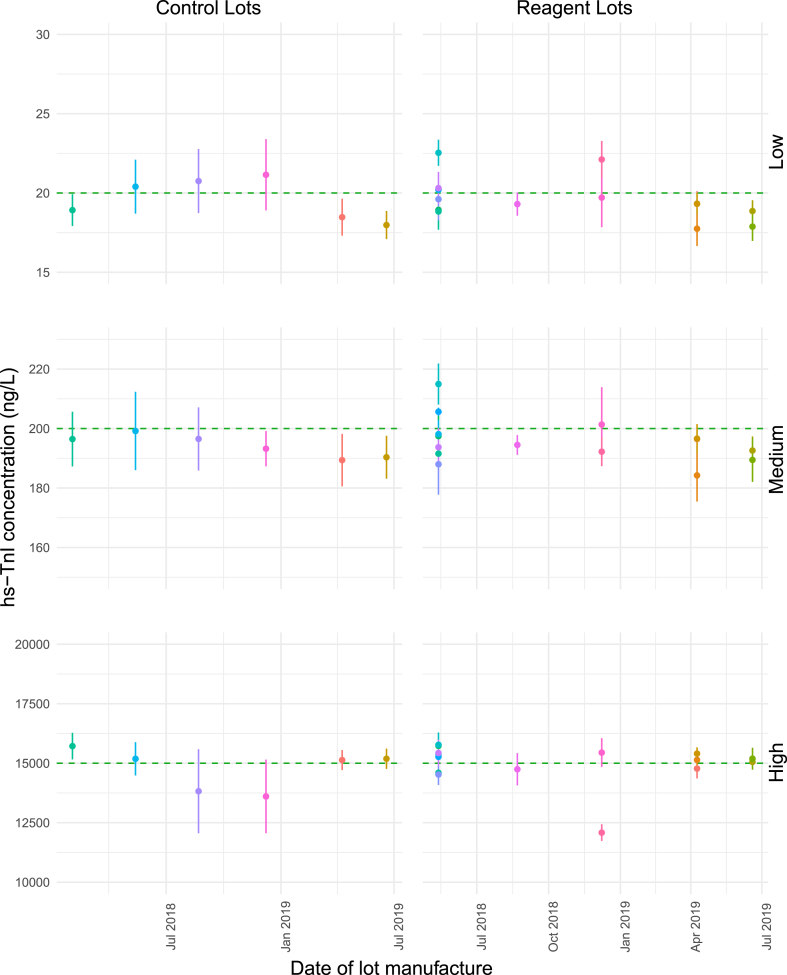
**Lot-specific mean hs-TnI concentrations over lot manufacturing date on the ARCHITECT i2000SR**. Points indicate lot-specific means. Error bars represent lot-specific standard deviations. Horizontal dashed line represents the target-release hs-TnI concentration.

To illustrate any reagent and control lot-specific bias and variability for each control concentration level, individual lot’s mean and standard deviations were plotted against the manufacturing date of the lots as shown in Fig. 1 for the ARCHITECT ci4100 data and in Fig. 2 for the ARCHITECT i2000SR data. The Alinity ci data was excluded from this analysis as it comprised only three lots.

### 3.2 The impact of lot age on hs-TnI measurements

**Fig. 3:**
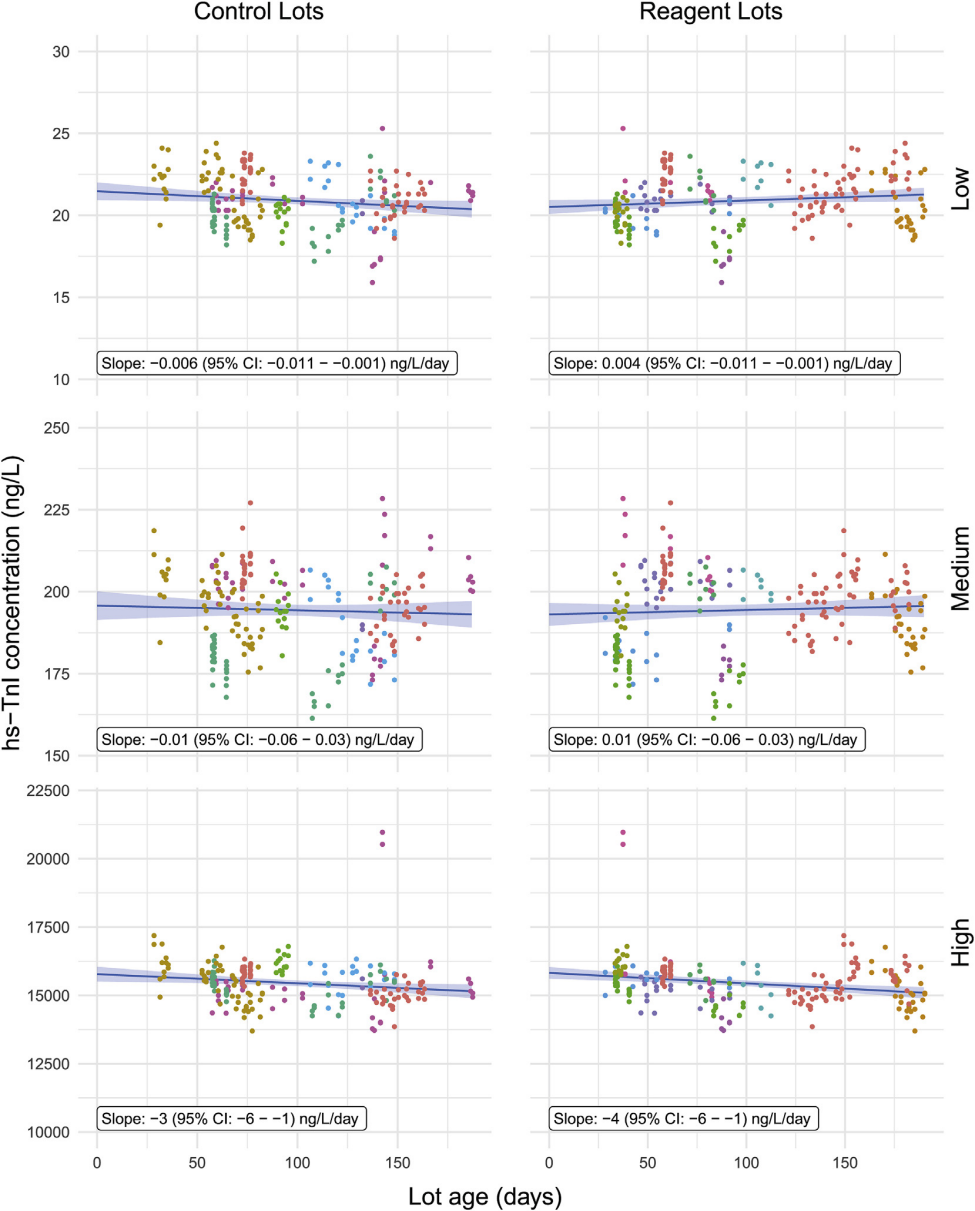
**hs-TnI concentration at three levels over the age of the controls and reagents on the ARCHITECT ci4000**. Regression lines represent marginal mean hs-TnI concentration against the margin of control age (left column) or reagent age (right column). Shaded bands represent 95% confidence intervals. Coloration of data points represent individual reagent lots.

**Fig. 4:**
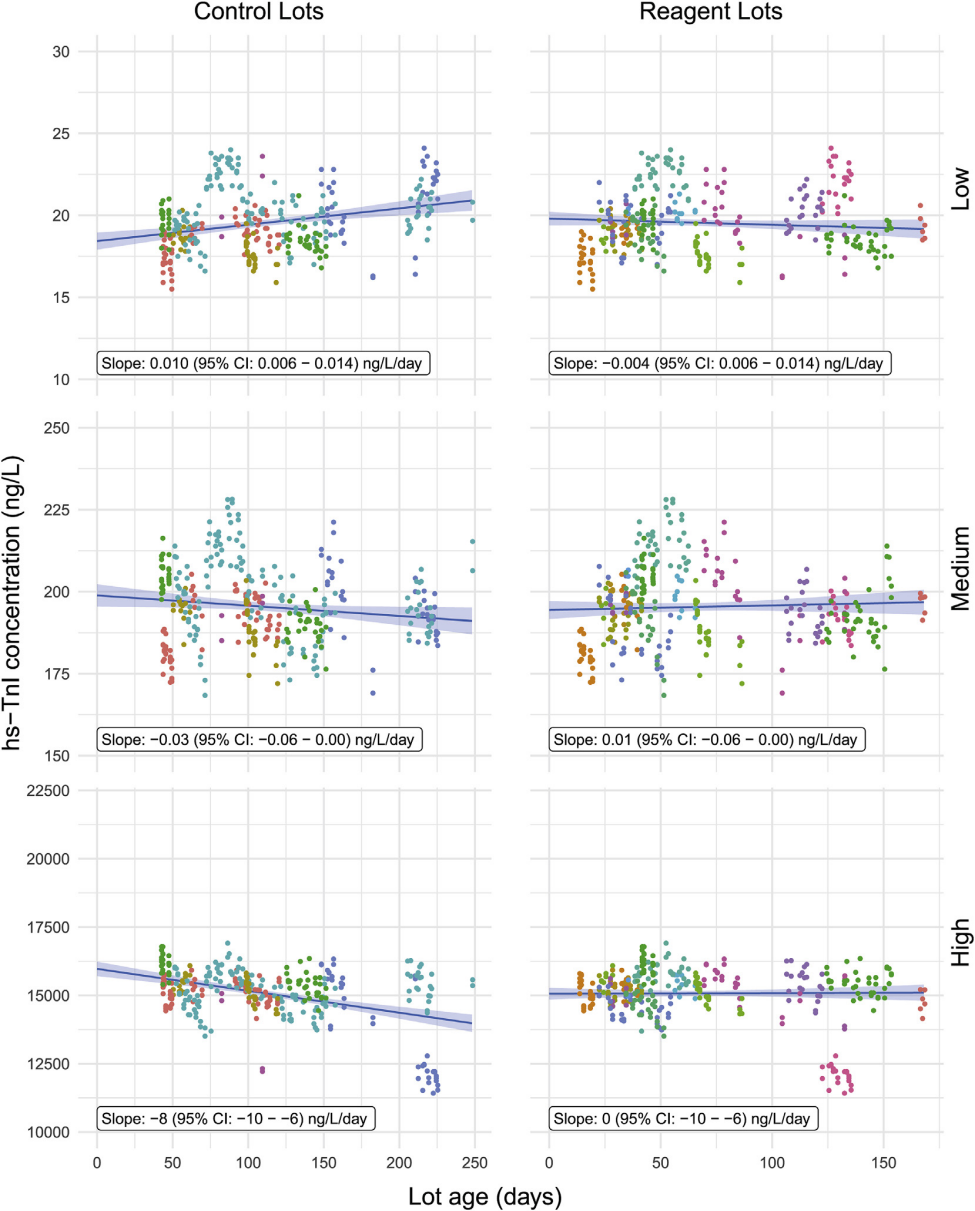
**hs-TnI concentration at three levels over the age of the controls and reagents on the ARCHITECT i2000SR**. Regression lines represent marginal mean hs-TnI concentration against the margin of control age (left column) or reagent age (right column). Shaded bands represent 95% confidence intervals. Coloration of data points represent individual lots.

The effect of control or reagent age on any change in mean hs-TnI measurements was evaluated using simple multivariable linear regression models with control age and reagent age as independent variables and historical QC data from the ARCHITECT ci4100 and ARCHITECT i2000SR for each QC concentration level as dependent variables. Mixed effects models and linear regression models with linear splines were tested but were considered inadequate due to sample size limitations. The marginal effects are shown in Fig. 3 for the ARCHITECT ci4100 and in Fig. 4 for the ARCHITECT i2000SR.

For this study we also evaluated variability between consecutive runs of the hs-TnI assay with the low concentration controls (20 ​ng/L). The differences between consecutive runs were evaluated across the entire historical dataset, grouped by reagents and control lots, and the instances where deviations were larger than 6 ​ng/L were counted. This value is considered by the European Society for Cardiology guidelines to be a critical shift in TnI between baseline and 1 ​h that may indicate a non-ST-elevation myocardial infarction in patients with onset of chest pain lasting more than 3 ​h [19].

The results can be found in Table S3 (Supplementary File 1) and show no consecutive shifts larger than 6 ​ng/L on any of the platforms. On three occasions QC outliers were flagged and QC was repeated after the initial error was identified. Subsequent results were within tolerated ranges.

### 3.3 Impact of time from calibration on hs-TnI measurements

**Fig. 5:**
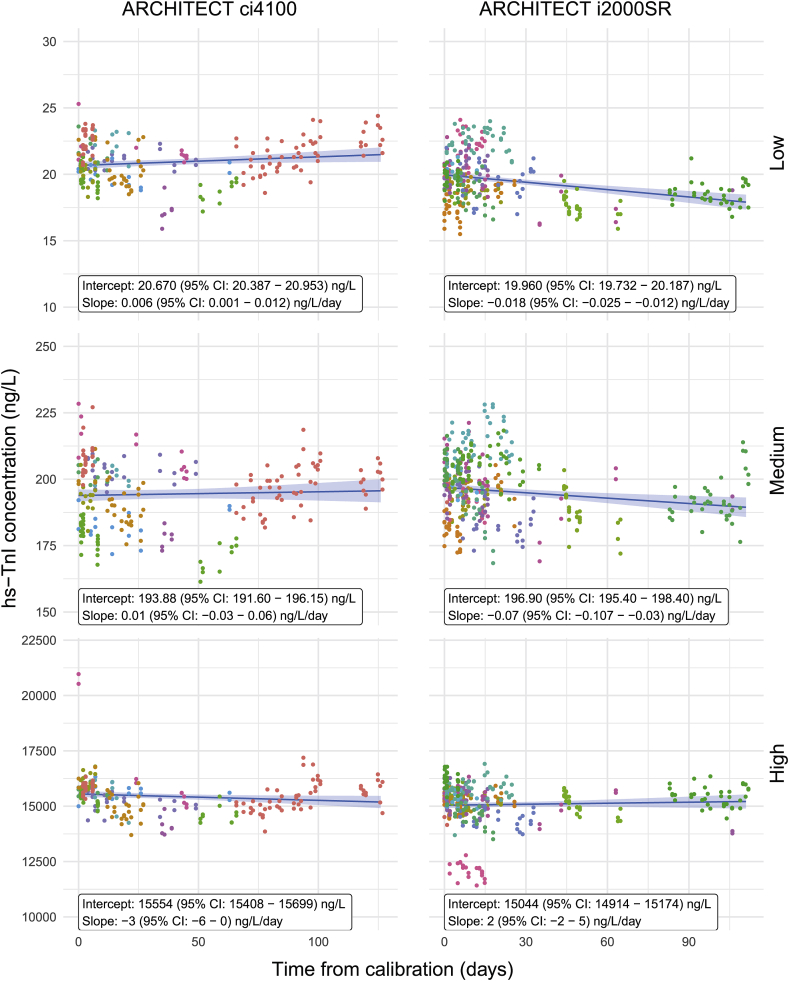
**hs-TnI concentration at three levels over the time from last calibration on the ARCHITECT ci4100 and ARCHITECT i2000SR**. Regression lines represent marginal mean hs-TnI concentration against the margin of time from last calibration. Shaded bands represent 95% confidence intervals. Coloration of data points represent individual reagent lots.

To evaluate whether time from last calibration of the ARCHITECT ci4100 and ARCHITECT i2000SR is associated with a change in mean hs-TnI measurement, simple univariate linear models were fitted to the historical measurement data over time from last calibration. The marginal effects are shown in Fig. 5 for both ARCHITECT analysers.

## 4 Discussion and limitations

The aim of the current study was to evaluate the analytical performance of the ARCHITECT and Alinity hs-TnI assay using retrospective data. The results for the three instruments provide evidence that QC material used in the five year’s testing range were not affected by systematic errors with respect to instrument performance or product variability, while showing performance stability over the age of the controls and reagents. Inter-lot CV’s were below manufacturer’s specifications (<10%) and both platforms exhibited similar trends in precision, indicating that the observed outcomes were probably not platform dependent but rather a reflection of product consistency.

Length of time between calibrations was shown to have a generally low impact on recovery to manufacturer’s targets, with consistency of measurements shown up to 100 days after calibration. However, for low concentrations, bias may somewhat increase after calibration intervals longer than 30 days but remained within manufacturer’s specifications. As per manufacturer’s instructions, calibration should be repeated every 30 days, for clinical purposes, providing a short calibration window to maximise the linearity of the assay. Additionally, the instruments were also calibrated when QC results varied by more than +/-2 standard deviations from the factory release testing values. This criterion was considered acceptable for results produced for research purposes in the CRC Core Lab. Nonetheless, the retrospective analysis performed in this study has resulted in a tightening of quality criteria for future use of the hs-TnI assay on the CRC Core Lab systems.

The European Society for Cardiology guidelines consider a >6 ​ng/L shift in TnI between baseline and 1 ​h to be indicative of a non-ST-elevation myocardial infraction in patients with onset of chest pain lasting more than 3 ​h [19]. This diagnostic algorithm relies on two key concepts: 1) the increase of the serum cardiac TnI concentration is associated with an increased probability of myocardial infraction, 2) early absolute change of this concentration over 1 ​h can be used as a surrogate measure for the absolute change over longer periods of three and 6 ​h and provide incremental diagnostic value to the TnI value at presentation. It is important to distinguish changes TnI concentration in patients from any variability introduced during measurement. Good laboratory practice is essential to keep measurement variability within acceptable limits for single sets of assay reagents, calibrators and quality control agents. We therefore evaluated the measurement variability of the hs-TnI assay at the low control level (20 ​ng/L) by recording >6 ​ng/L changes between consecutive runs within control and reagent lots. Across the 5-year historical dataset no instances of consecutive >6 ​ng/L shifts were found.

This study represents and independent QC validation study and was conducted on data collected from a single site over a relatively long period of time. The results from this retrospective performance study may inform biomedical and research laboratories on the long-term performance of the hs-TnI assay materials produced by Abbott Laboratories. It may also inform the manufacturers on the production of these materials and the guidelines for their use. The study can also drive decisions around quality control regimes in laboratories and guide their own performance analysis.

Only controls from the vendor were tested in this study. Future work will focus on utilizing third party controls, as well as controls with concentrations below 20 ​ng/L. Validating the performance of the hs-TnI assay at lower concentrations may provide insight into its application for diagnosis within asymptomatic patient cohorts and healthier populations.

### 4.1 Limitations

Only controls from the vendor were tested in this study. Future work will focus on utilizing third party controls, as well as controls with concentrations below 20 ​ng/L. Validating the performance of the hs-TnI assay at lower concentrations may provide insight into its application for diagnosis within asymptomatic patient cohorts and healthier populations.

## 5 Conclusion

Assay reagents and quality controls for the hs-TnI assay on the ARCHITECT i2000sr and ARCHITECT ci4000 systems have consistent and age-independent performance characteristics over the 5 years that they have been used in the Core Lab Facility in the University College Dublin Clinical Research Centre. The Alinity ci System exhibits similar performance characteristics. Independent QC and QC using patient materials in future studies may provide further details on the performance of the assays in this study.

## Funding

Funded by fellowship funding from Abbott Laboratories.

## Author statement

Conceptualisation: Rosanna Inzitari, Sebastian Vencken, David Daghfal, Marie Galligan, Peter Doran.

Data curation: Rosanna Inzitari, Sebastian Vencken, David Daghfal, Karl McAuley, Anthony McDermott.

Formal analysis: Sebastian Vencken.

Funding acquisition: David Daghfal, Peter Doran.

Investigation: Rosanna Inzitari, Sebastian Vencken, David Daghfal, Karl McAuley, Anthony McDermott.

Methodology: Rosanna Inzitari, Sebastian Vencken, David Daghfal.

Project administration: Rosanna Inzitari.

Resources: Rosanna Inzitari, David Daghfal, Peter Doran.

Software: Sebastian Vencken.

Supervision: Rosanna Inzitari, David Daghfal, Peter Doran.

Validation: Rosanna Inzitari, Sebastian Vencken.

Visualization: Sebastian Vencken.

Roles/Writing - original draft: Rosanna Inzitari, Sebastian Vencken, David Daghfal.

Writing - review & editing: Rosanna Inzitari, Sebastian Vencken, David Daghfal.

## Declaration of competing interest

David Daghfal is an employee of Abbott Laboratories. Professor Peter Doran is in receipt of an educational grant from 10.13039/100014386Abbott Diagnostics to support Research Fellowship in biomarker analysis. Rosanna Inzitari, Sebastian Vencken, Karl McAuley, Anthony McDermott and Marie Galligan have no interests to declare.

## Appendix A Supplementary data

Multimedia component 1 Supplementary File 1: Supplementary methods and tables. Multimedia component 1

Multimedia component 2 Supplementary File 2: R code used for analysis of all study data. In R Markdown format. Multimedia component 2
