# Supplementary material for: Time-related performance characteristics of high-sensitivity troponin I assay using manufacturer’s controls and reagents
Source: Pract Lab Med. 2021 Mar 26;25:e00217. doi: 10.1016/j.plabm.2021.e00217 (PMC8058562; doi:10.1016/j.plabm.2021.e00217)
Supplement: Multimedia component 1 — Supplementary File 1: Supplementary methods and tables. [file mmc1.docx]

**Supplementary Methods**

**Equations for regression models used.**

TnI concentration and lot age:

${TnI concentration}_{i}=\beta_{0}+\beta_{1}{Control lot age}_{i}+ \beta_{2}{Reagent lot age}_{i}+\varepsilon_{i}$ Eq. (A.1)

TnI concentration and time from last calibration:

${TnI concentration}_{i}=\beta_{0}+\beta_{1}{Calibration age}_{i}+\varepsilon_{i}$ Eq. (A.2)

For both models:

$$\varepsilon_{i}\sim N(0, \sigma_{e}^{2})$$

**Supplementary Tables**

**Table S1:** Lot serial numbers of quality control lots and reagents lots used in this study. The reagents lots shaded in red were recalled (see Supplementary File 3 for the Abbott field safety notice).

| **ARCHITECT ci4100** | | **ARCHITECT i2000SR** | | **Alinity ci** | |
| --- | --- | --- | --- | --- | --- |
| **Quality Control Lot #** | **Reagents Lot #** | **Quality Control Lot #** | **Reagents Lot #** | **Quality Control Lot #** | **Reagents Lot #** |
| 72088UI00 | 84147UI00 | 04915UI00 | 06388UI00 | 95249UI00 | 96031UI00 |
| 69042UI00 | 76003UI00 | 02429UI00 | 05246UI00 | 96307UI00 | 02903UI00 |
| 66393UI00 | 73352UI00 | 91174UI00 | 01122UI00 | 05921UI00 | 06332UI00 |
| 62385UI00 | 72203UI00 | 94347UI00 | 04458UI00 |  |  |
| 60246UI00 | 69457UI00 | 88303UI00 | 03266UI00 |  |  |
| 59148UI00 | 67067UI00 | 84131UI00 | 94158UI00 |  |  |
| 54247UI00 | 58927UI00 | 04918UI00 | 93555UI00 |  |  |
|  | 65367UI00 |  | 92013UI00 |  |  |
|  | 63187UI00 |  | 91126UI00 |  |  |
|  | 62026UI00 |  | 90421UI00 |  |  |
|  | 60103UI00 |  | 90099UI00 |  |  |
|  | 55946UI00 |  | 89242UI00 |  |  |
|  | 54938UI00 |  | 88511UI00 |  |  |
|  |  |  | 88368UI00 |  |  |
|  |  |  | 84147UI00 |  |  |
|  |  |  | 07261UI00 |  |  |

**Table S2:** Descriptive statistics for hs-TnI reagent lots and control lots on ARCHITECT ci4100, ARCHITECT i2000SR and Alinity ci, limited to results from lots that are <128 days old.

| **ARCHITECT ci4100** | | | |
| --- | --- | --- | --- |
| **Statistic** | **Level 1 (20 ng/L)** | **Level 2 (200 ng/L)** | **Level 3 (15000 ng/L)** |
| N_Reagent_ | 7 | 7 | 7 |
| N_Control_ | 5 | 5 | 5 |
| N_Total_ | 157 | 156 | 156 |
| Mean *ng/L* | 20.60 | 195.9 | 15683 |
| Bias_Target_ *%* | 3.02 | -2.07 | 4.55 |
| Bias_FRT_ *%* | 2.26 | 1.37 | -0.40 |
| CV_Intrareagents_ *%* | 7.63 | 3.64 | 2.93 |
| CV_Interreagents_ *%* | 5.43 | 5.58 | 2.72 |
| CV_Intracontrol_ *%* | 7.66 | 3.79 | 2.92 |
| CV_Intercontrol_ *%* | 5.66 | 6.13 | 2.67 |
| CV_Total_ *%* | 8.93 | 6.65 | 3.89 |
| **ARCHITECT i2000SR** | | | |
| **Statistic** | **Level 1 (20 ng/L)** | **Level 2 (200 ng/L)** | **Level 3 (15000 ng/L)** |
| N_Reagent_ | 12 | 12 | 12 |
| N_Control_ | 5 | 5 | 5 |
| N_Total_ | 215 | 216 | 216 |
| Mean *ng/L* | 19.40 | 196.5 | 15297 |
| Bias_Target_ *%* | -2.99 | -1.77 | 1.98 |
| Bias_FRT_ *%* | 0.40 | -0.08 | -0.87 |
| CV_Intrareagents_ *%* | 4.82 | 3.90 | 2.81 |
| CV_Interreagents_ *%* | 8.14 | 4.23 | 6.33 |
| CV_Intracontrol_ *%* | 7.09 | 5.19 | 3.76 |
| CV_Intercontrol_ *%* | 7.27 | 3.56 | 5.97 |
| CV_Total_ *%* | 9.65 | 6.31 | 4.42 |
| **Alinity ci** | | | |
| **Statistic** | **Level 1 (20 ng/L)** | **Level 2 (200 ng/L)** | **Level 3 (15000 ng/L)** |
| N_Reagent_ | 3 | 3 | 3 |
| N_Control_ | 3 | 3 | 3 |
| N_Total_ | 108 | 108 | 108 |
| Mean *ng/L* | 19.94 | 198.7 | 15383 |
| Bias_Target_ *%* | -0.31 | -0.67 | 2.55 |
| Bias_FRT_ *%* | -4.37 | -0.94 | 2.37 |
| CV_Intrareagents_ *%* | 6.43 | 4.59 | 4.02 |
| CV_Interreagents_ *%* | 3.12 | 3.34 | 4.22 |
| CV_Intracontrol_ *%* | 6.34 | 4.55 | 3.94 |
| CV_Intercontrol_ *%* | 3.49 | 3.36 | 4.27 |
| CV_Total_ *%* | 6.78 | 5.39 | 5.26 |

**Table S3:** Frequency and proportion of comparisons between consecutive runs that are >6 ng/L (Low concentration (20 n/L) data only). The reagents lots shaded in red were recalled.

| **ARCHITECT ci4100** | | | | |
| --- | --- | --- | --- | --- |
| **Quality Control Lot #** | **Reagents Lot #** | **N with >6 ng/L consecutive difference** | **Total N** | **%** |
| 54247UI00 | 54938UI00 | 0 | 57 | 0 |
| 59148UI00 | 54938UI00 | 0 | 24 | 0 |
| 59148UI00 | 55946UI00 | 0 | 28 | 0 |
| 60246UI00 | 58927UI00 | 0 | 6 | 0 |
| 60246UI00 | 60103UI00 | 1 | 54 | 1.85 |
| 60246UI00 | 62026UI00 | 0 | 14 | 0 |
| 62385UI00 | 63187UI00 | 0 | 34 | 0 |
| 62385UI00 | 65367UI00 | 0 | 8 | 0 |
| 66393UI00 | 67067UI00 | 0 | 8 | 0 |
| 66393UI00 | 69457UI00 | 0 | 12 | 0 |
| 69042UI00 | 69457UI00 | 0 | 2 | 0 |
| 72088UI00 | 72203UI00 | 0 | 20 | 0 |
| 72088UI00 | 73352UI00 | 0 | 6 | 0 |
| 72088UI00 | 76003UI00 | 0 | 12 | 0 |
| **ARCHITECT i2000SR** | | | | |
| **Quality Control Lot #** | **Reagents Lot #** | **N with >6 ng/L consecutive difference** | **Total N** | **%** |
| 02429UI00 | 01122UI00 | 0 | 6 | 0 |
| 02429UI00 | 03266UI00 | 0 | 33 | 0 |
| 02429UI00 | 04458UI00 | 0 | 14 | 0 |
| 02429UI00 | 05246UI00 | 0 | 14 | 0 |
| 04915UI00 | 05246UI00 | 0 | 6 | 0 |
| 04915UI00 | 06388UI00 | 0 | 37 | 0 |
| 84131UI00 | 84147UI00 | 0 | 54 | 0 |
| 88303UI00 | 84147UI00 | 0 | 6 | 0 |
| 88303UI00 | 88368UI00 | 0 | 23 | 0 |
| 88303UI00 | 88511UI00 | 0 | 36 | 0 |
| 88303UI00 | 89242UI00 | 0 | 10 | 0 |
| 88303UI00 | 90099UI00 | 0 | 2 | 0 |
| 88303UI00 | 90421UI00 | 0 | 28 | 0 |
| 88303UI00 | 91126UI00 | 0 | 22 | 0 |
| 88303UI00 | 92013UI00 | 0 | 6 | 0 |
| 88303UI00 | 93555UI00 | 0 | 2 | 0 |
| 91174UI00 | 92013UI00 | 0 | 1 | 0 |
| 91174UI00 | 93555UI00 | 0 | 21 | 0 |
| 91174UI00 | 94158UI00 | 0 | 19 | 0 |
| 94347UI00 | 93555UI00 | 0 | 2 | 0 |
| **Alinity ci** | | | | |
| **Quality Control Lot #** | **Reagents Lot #** | **N with >6 ng/L consecutive difference** | **Total N** | **%** |
| 05921UI00 | 06332UI00 | 0 | 48 | 0 |
| 95249UI00 | 96031UI00 | 0 | 25 | 0 |
| 96307UI00 | 02903UI00 | 0 | 33 | 0 |
| 96307UI00 | 96031UI00 | 0 | 2 | 0 |
